# Comparative genome analysis and genome evolution of members of the magnaporthaceae family of fungi

**DOI:** 10.1186/s12864-016-2491-y

**Published:** 2016-02-25

**Authors:** Laura H. Okagaki, Joshua K. Sailsbery, Alexander W. Eyre, Ralph A. Dean

**Affiliations:** Center for Integrated Fungal Research, North Carolina State University, 851 Main campus Drive, Raleigh, NC 27606 USA; Bayer CropScience, Research Triangle Park, 2 TW Alexander Drive, Durham, NC 27709 USA; Department of Plant Pathology, North Carolina State University, Raleigh, NC USA

**Keywords:** Magnaporthaceae, Magnaporthe, Gaeumannomyces, Two-speed genome, Zig zag model, Comparative genomics, CAzymes, Transcription factors, Diversifying selection, Purifying selection

## Abstract

**Background:**

Magnaporthaceae, a family of ascomycetes, includes three fungi of great economic importance that cause disease in cereal and turf grasses: *Magnaporthe oryzae* (rice blast), *Gaeumannomyces graminis* var. *tritici* (take-all disease), and *Magnaporthe poae* (summer patch disease). Recently, the sequenced and assembled genomes for these three fungi were reported. Here, the genomes were compared for orthologous genes in order to identified genes that are unique to the Magnaporthaceae family of fungi. In addition, ortholog clustering was used to identify a core proteome for the Magnaporthaceae, which was examined for diversifying and purifying selection and evidence of two-speed genome evolution.

**Results:**

A genome-scale comparative study was conducted across 74 fungal genomes to identify clusters of orthologous genes unique to the three Magnaporthaceae species as well as species specific genes. We found 1149 clusters that were unique to the Magnaporthaceae family of fungi with 295 of those containing genes from all three species. Gene clusters involved in metabolic and enzymatic activities were highly represented in the Magnaporthaceae specific clusters. Also highly represented in the Magnaporthaceae specific clusters as well as in the species specific genes were transcriptional regulators. In addition, we examined the relationship between gene evolution and distance to repetitive elements found in the genome. No correlations between diversifying or purifying selection and distance to repetitive elements or an increased rate of evolution in secreted and small secreted proteins were observed.

**Conclusions:**

Taken together, these data show that at the genome level, there is no evidence to suggest multi-speed genome evolution or that proximity to repetitive elements play a role in diversification of genes.

**Electronic supplementary material:**

The online version of this article (doi:10.1186/s12864-016-2491-y) contains supplementary material, which is available to authorized users.

## Background

Genome comparison studies have become critical to understanding the evolutionary relationships between similar species. Genome sequencing and expression data have become more cost-effective and easier to generate, resulting in an increase in the number of available genomes for analysis. In mycology, many of the genomes are poorly annotated, resulting in a need for large scale genome analysis to identify genes that have similar function. For pathogens, comparisons can help to find novel drug targets, mechanisms of infection, or common genes that might shed light on pathogenic and non-pathogenic lifestyles.

Homologs are genes that are shared among related organisms and can be used for genome comparisons. Homologs can fall into two different subclasses: orthologs and paralogs. Orthologs are derived from a common ancestor but usually diverge by speciation, resulting in retention of similar functions during evolution. In contrast, paralogs typically diverge after speciation and are the result of gene duplication events and may or may not retain similar functions. Orthologs and paralogs can be useful tools in genome comparison studies because they can highlight genes shared among species that are important to conserved biological processes or can reveal those genes that are unique to a particular subset of fungi, such as families of fungi or fungi with a specific lifestyle. Several algorithms have been developed to study orthologs across species, but most are limited to comparisons between only two species. OrthoMCL [1] is an algorithm used for the identification of orthologs between multiple species. Developed by Li et al. [2], OrthoMCL uses multiple steps including BLASTp and Markov clustering in order to group genes into likely orthologous clusters. Using such algorithms, genes with similar functions as well as those genes unique to each species can be identified.

The Magnaporthaceae family of fungi contains several economically important plant pathogens. Among the pathogenic members of this family are *Magnaporthe oryzae*, *Gaeumannomyces graminis* var. *tritici*, and *Magnaporthe poae. M. oryzae* is known as the rice blast fungus and causes disease in rice, wheat, and barley following landing of conidia on the host plant leaf [3, 4]. Upon germination on the hydrophobic leaf surface, the formation of a specialized infection structure, the appressorium, is stimulated. The appressorium penetrates the leaf surface allowing the fungus to invade and spread in the plant tissue. *M. oryzae* outbreaks have been known to devastate vast acreages of rice on a regular basis and is a major concern for global food security [4, 5]. More recently, *M. oryzae* has also been shown to cause disease on other cultivated grasses including barley and wheat, increasing its threat to the food supply [3, 6]. *G. graminis* var. *tritici* is the causative agent of take-all disease in wheat [3, 7]. Unlike *M. oryzae*, which targets the leaf of the plant, *G. graminis* var. *tritici* attacks the roots of wheat plants resulting in root rot. Hyphae of the soil-borne fungus wrap around the root and invade the root structure causing tissue necrosis and subsequent killing of the plant [3, 7]. *M. poae*, the causative agent of summer patch disease in turf grasses, acts in a similar manner to *G. graminis* var. *tritici* and attacks the roots of grasses causing root-rot and subsequent host plant death [3].

Identification of proteins that are involved with host-pathogen interactions has, until recently, relied on molecular biology techniques at the bench. For plant pathogens, several classes of proteins are frequent targets of further study including carbohydrate active enzymes (CAzymes), transcriptional regulators, and secreted proteins. CAzymes can be classified into six subsets [8]: auxiliary activity (AA), carbohydrate binding molecules (CBM), carbohydrate esterases (CE), glycoside hydrolases (GH), glycosyltransferases (GT), and polysaccharide lyases (PL). Comparative studies of CAzymes in 103 fungal proteomes were performed by Zhao et al. [9], and showed for *M. oryzae*, *G. graminis* var. *tritici*, and *M. poae* that GHs were the most abundant class. Targets of GHs include cellulose, glycans, glucans, and chitin, suggesting both plant and fungal targets for this enzyme class [8, 9].

Fungal effector proteins are secreted proteins, often less than 250 amino acids in length, which interact with host plant proteins in order to modulate the host immune system and promote infection [10, 11]. Effectors proteins have been shown to be highly diversifying [3, 6, 11–24] and may be undergoing accelerated evolution. Studies in *M. oryzae* have shown that some effector proteins are undergoing high rates of diversification in order to evade the host immune response, suggesting that there is selection pressure by the host environment to rapidly accumulate non-synonymous mutations [3, 12, 15, 21, 22, 24, 25]. These data suggest that diversification of genes through mutation is one mechanism for fungi to evolve to escape plant recognition. This concept of two-speed genome evolution, where virulence genes evolve more rapidly than other genes, has implicated repetitive DNA elements, including retrotransposons, in the increased rate of evolution in effector proteins [12–22]. Together, CAzymes and small secreted proteins are critical to initial host-pathogen interactions that allow a fungal pathogen to degrade and enter host cells while modulating their response to invasion. With more recent advances in bioinformatics, both CAzymes and small secreted proteins of special interest can be identified and characterized prior to studying them at the bench.

The goal of this study was two-fold: identify genes and gene clusters that are unique to the Magnaporthaceae family of fungi in order to identify genes that may be involved pathogenesis, and identify a core proteome of conserved genes and identify functional clusters that are undergoing rapid diversification. First, the protein sequences from 74 fungal genomes, including the genomes of *M. oryzae*, *G. graminis* var. *tritici*, and *M. poae*, were chosen from the Broad Institute’s Fungal Genome Initiative [26] for OrthoMCL analysis. The genomes included consisted of plant and animal pathogens as well as the genomes of model fungi, such as *Saccharomyces cerevisiae*. OrthoMCL clusters that contained only genes from the Magnaporthaceae family of fungi and unclustered genes that are species specific were further analyzed. Gene Ontology annotation (GO annotation) [27], and InterProScan [28] protein domain identification were used to determine the putative functions for each cluster of orthologs. We hypothesized that genes and gene clusters involved with metabolic process would be highly represented in the Magnaporthaceae specific and species specific genes. The data suggests, however, that proteins with enzymatic function and transcriptional regulators were highly represented in orthologous clusters that are unique to the Magnaporthaceae. In addition, we used Hmmscan [29, 30], to identify Magnaporthaceae specific clusters and species specific “unique” genes that had putative CAzyme function. We found that few CAzymes were clustered by OrthoMCL, while a higher number were identified in the species specific genes.

Second, OrthoMCL clusters containing at least one gene from each of the three Magnaporthaceae species were identified as the “core proteome”. We hypothesized that secreted proteins and specifically secreted proteins with enzymatic and protease functions would be undergoing diversifying selection. In addition, we hypothesized that genes under diversifying selection would be closer to repetitive elements than genes that are under neutral or purifying selection. Phylogenetic Analysis by Maximum Likelihood (PAML) [31] was used to identify genes that exhibited purifying selection or diversifying selection and compared to repetitive element locations in the genome. Additionally, secreted proteins were identified using TargetP [32] and SignalP [33] and were examined for their proximity to repetitive elements. Surprisingly, the data suggests that there is no correlation between genes undergoing diversifying selection or genes with higher mutations rates and distance to repetitive elements. In addition, we found no evidence that secreted proteins are subjected to more diversifying selection than purifying selection. Taken together, we found no evidence of two-speed genome evolution between the three Magnaporthaceae species examined.

## Results

### Ortholog clustering

Ortholog clustering can be used to identify important patterns in gene conservation across diverse organisms such as the fungal kingdom when comparing a large number of eukaryotic genomes. Clustering can also reveal unique sets of genes that are important to one species or group of fungi that are not found in other species. In order to identify genes that are unique to and shared among the three Magnaporthaceae species (*M. oryzae*, *M. poae*, and *G. graminis* var. *tritici*), we utilized an ortholog clustering algorithm. Seventy-four fungal genomes [34] were used to for ortholog identification using OrthoMCL [1, 2]. The genomes used represented human pathogens, plant pathogens, model organisms, and environmental fungi (Additional file 1, Additional file 2), and represented four phyla and twelve classes of fungi. These genomes were chosen for OrthoMCL analysis because all genomes were sequenced, assembled and annotated using a similar work-flow by the Broad Institute. 12,991 protein sequences were analyzed for *M. oryzae*, while 14,650 and 12,329 were analyzed for *G. graminis* var. *tritici* and *M. poae* respectively (Table 1). Approximately 22–25 % of Magnaporthaceae species genes were removed from clustering analysis after BLASTp alignment. An additional 5–10 % of Magnaporthaceae genes were either not clustered with any other genes or were clustered with genes from a single species. These genes, along with the gene removed after BLASTp analysis were combined to create the “unique gene” category (Table 1).

**Table 1:** OrthoMCL and unique gene summary

	Input genes	Clustered genes	% Clustered	Unclustered	Clustered as single species	Total unique genes	% Unique
*M. oryzae*	12991	9723	76.58	3268	1392	4660	35.87
*G. graminis*	14650	10709	75.12	3941	1059	5000	34.13
*M. poae*	12329	9518	78.58	2811	593	3404	27.61

Protein sequences from 74 fungal genomes including *M. oryzae*, *G. graminis* var. *tritici*, and *M. poae,* were used for OrthoMCL orthologous gene clustering (Input Genes). During OrthoMCL analysis, some genes were eliminated during all-against-all BLASTp (Unclustered), others were not clustered with other genes or were only clustered with genes within in single species (Clustered as Single Species). Unclustered and genes clustered as a single species were combined (Total Unique Genes)

**Fig. 1:**
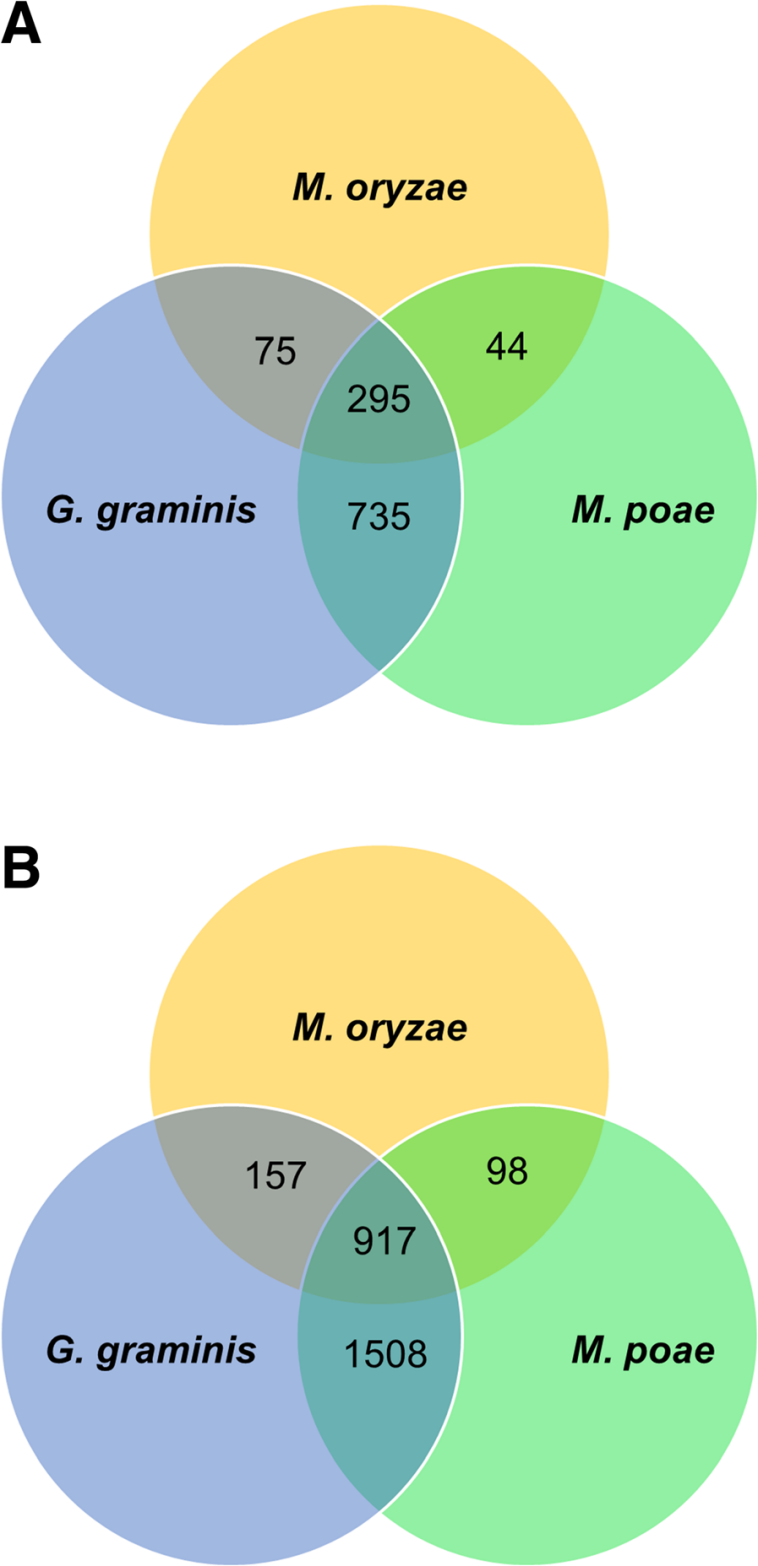
OrthoMCL summary. Orthologs for 74 fungal genomes were clustered using OrthoMCL. **a** Clusters containing only genes from the Magnaporthaceae species were identified and counted. **b** Genes contained in clusters from each category were quantified

The analysis of the 74 fungal genomes resulted in a total of more than 43,000 clusters representing more than 572,000 genes (Table 2, Additional file 3). Approximately 76.6 % of *M. oryzae* sequences, 75.1 % of *G. graminis* var. *tritici* protein sequences, and 78.6 % of *M. poae* protein sequences were clustered during OrthoMCL analysis (Table 1). Of the ortholog clusters, 1149 clusters were specific to the Magnaporthaceae species (Table 2, Fig. 1a). The Magnaporthaceae specific clusters represented 2680 genes (Table 2, Fig. 1b). Two-hundred ninety five clusters contained genes from all three Magnaporthaceae species, and represented 917 genes. *M. poae* and *G. graminis* var. *tritici* shared the most clusters, 735 clusters containing 1508 genes, while *M. oryzae* and *M. poae* shared the fewest with 44 shared clusters containing 98 genes. Taken together, these data suggest that *G. graminis* var. *tritici* and *M. poae* are more closely related than are *M. poae* and *M. oryzae*, or *G. graminis* var. *tritici* and *M. oryzae*. These data support previous findings by Luo et al. [35] and Okagaki et al. [36], which showed using phylogenetic and syntenic analysis that *M. poae* and *G. graminis* var. *tritici* are more closely related.

**Table 2:** Magnaporthaceae specific OrthoMCL cluster summary

	Clusters	Genes
Total	43172	572694
Magnaporthaceae	1149	2680

OrthoMCL was used to cluster 74 fungal genome. Clusters containing single genes or genes from a single species were eliminated (Clusters). Those clusters containing genes from *M. oryzae*, *G. graminis* var. *tritici*, and *M. poae* and no other species were identified

### Cluster function identification

**Fig. 2:**
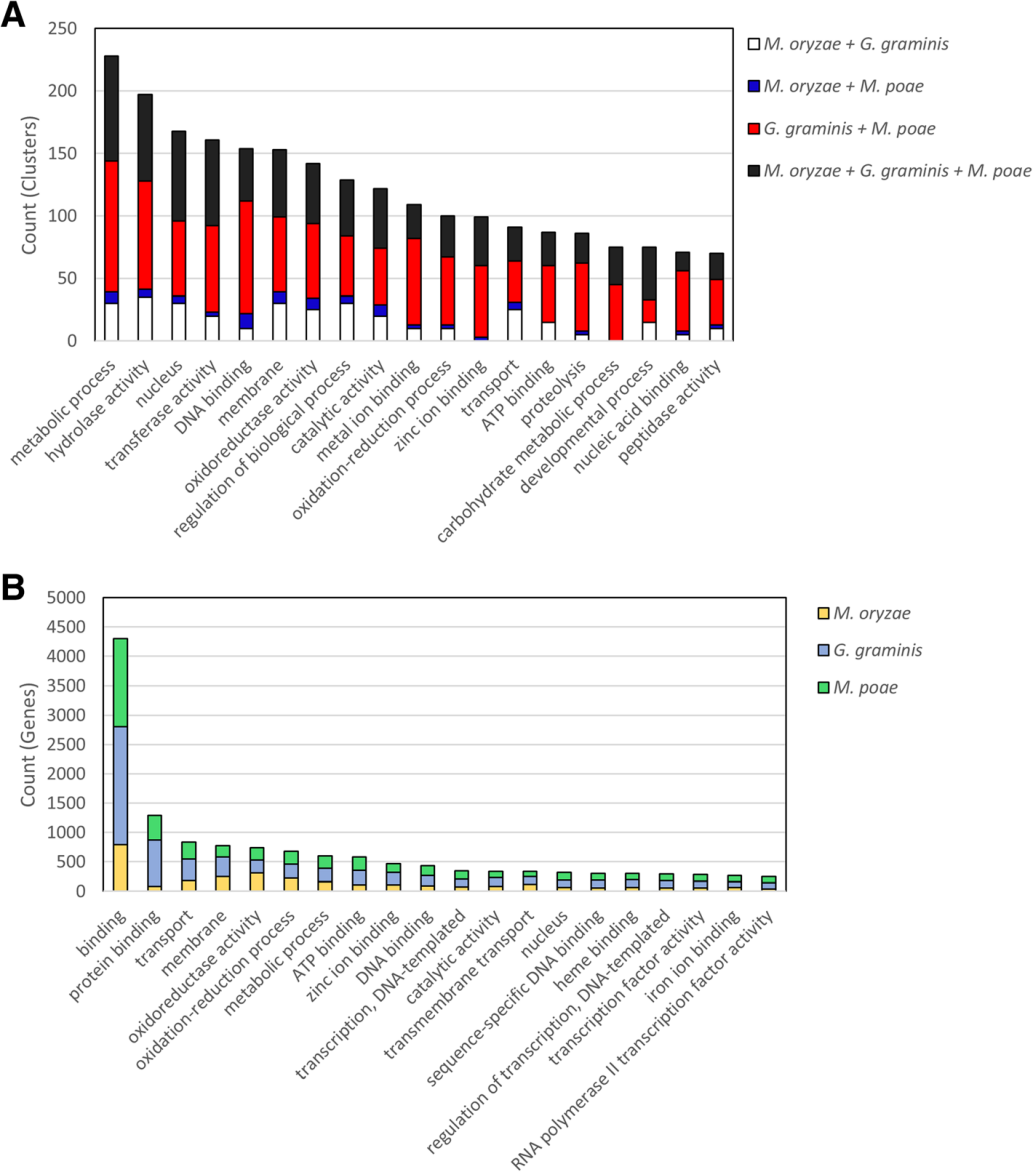
Putative functions of orthologs and unique genes. **a** Orthologous clusters from OrthoMCL that contained only Magnaporthaceae family genes were analyzed using Blast2GO. Putative functions based on Gene Ontology (GO) annotation. The most abundant GO categories were graphed. **b** Genes that were not clustered by OrthoMCL or genes in clusters that contained a single species were considered unique genes for each species. Putative functions were identified using GO annotation within InterProScan. The most abundant 20 GO categories were graphed

In order to identify the types of genes that are conserved and shared among the Magnaporthaceae, Blast2Go software suite [37] was used which included Gene Ontology annotation [27], *Aspergillus* Slim [27], and InterProScan [28] protein domain search functions to identify the functions of 1149 gene clusters identified in the OrthoMCL analysis.Three-hundred thirty nine unique functional categories were identified and the twenty most abundant functional categories were graphed (Fig. 2a). Of the 2680 genes analyzed using Blast2GO, 1746 genes that were clustered as orthologs have no known function (data not shown). The remaining 934 genes were used to identify putative cluster functions for the Magnaporthaceae specific OrthoMCL clusters. Six of the most abundant categories were genes involved in enzymatic activity, including metabolic process (228 genes), hydrolase activity (197 genes), transferase activity (161 genes), proteolysis (86 genes), and peptidase activity (70 genes). Categories that include nuclear localization and DNA binding were also common with 168 and 154 genes, respectively. Metal ion binding and zinc ion binding were both identified as putative functions in a large number of clusters (109 and 99, respectively). However, it was unclear if the ion binding activity was associated with transcriptional activity or other cellular processes. Taken together, these data suggest that transcriptional regulators, including transcription factors, are abundant among genes unique to the Magnaporthaceae family of fungi.

To determine putative functions for genes unique to each of the three Magnaporthaceae species, InterProScan was used to identify functional protein domains and GO annotations for each gene. One-hundred ninety four unique GO annotations were identified, with 244 genes returning no known protein domains and no GO annotation (Fig. 2b). Protein binding and other binding functions were highly represented in the unique proteins, with 4298 and 1290 genes represented by these two categories, respectively. Similar to the clustered genes, proteins with predicted enzymatic activity, including metabolic process (599 genes) and oxidoreductase activity (739 genes) were abundant in genes unique to each fungus. Additionally, six categories that were the most abundant were functions involved in transcription and transcriptional regulation, including DNA binding (437 genes) and transcription factor activity (281 genes). Again, similar to the Magnaporthaceae specific clusters, ion binding activity, with zinc (467 genes), heme (298 genes), and iron binding (266 genes) functions appeared in the most abundant twenty functional categories. Together with the Magnaporthaceae shared cluster data, these data suggest that proteins with enzymatic functions and transcriptional regulation proteins may be undergoing higher rates of mutation than genes with other functions.

### CAZyme identification and analysis

Fungal plant pathogens utilize a wide variety of carbohydrate active enzymes (CAZymes) in order to infect the host plant [9]. Previous analysis of a variety of fungal species showed that even mammalian commensal fungi retain an array of CAZymes [9]. There are six major classifications of CAZymes [8]: polysaccharide lyases (PL), glycosyltransferases (GT), glycoside hydrolases (GH), carbohydrate esterases (CE), carbohydrate binding molecules (CBM), and auxiliary activities (AA). Additional analysis showed that monocot pathogens, including *M. oryzae*, *G. graminis* var. *tritici*, and *M. poae*, exhibited an abundance of glycoside hydrolases and low numbers of polysaccharide lyases [9].

**Fig. 3:**
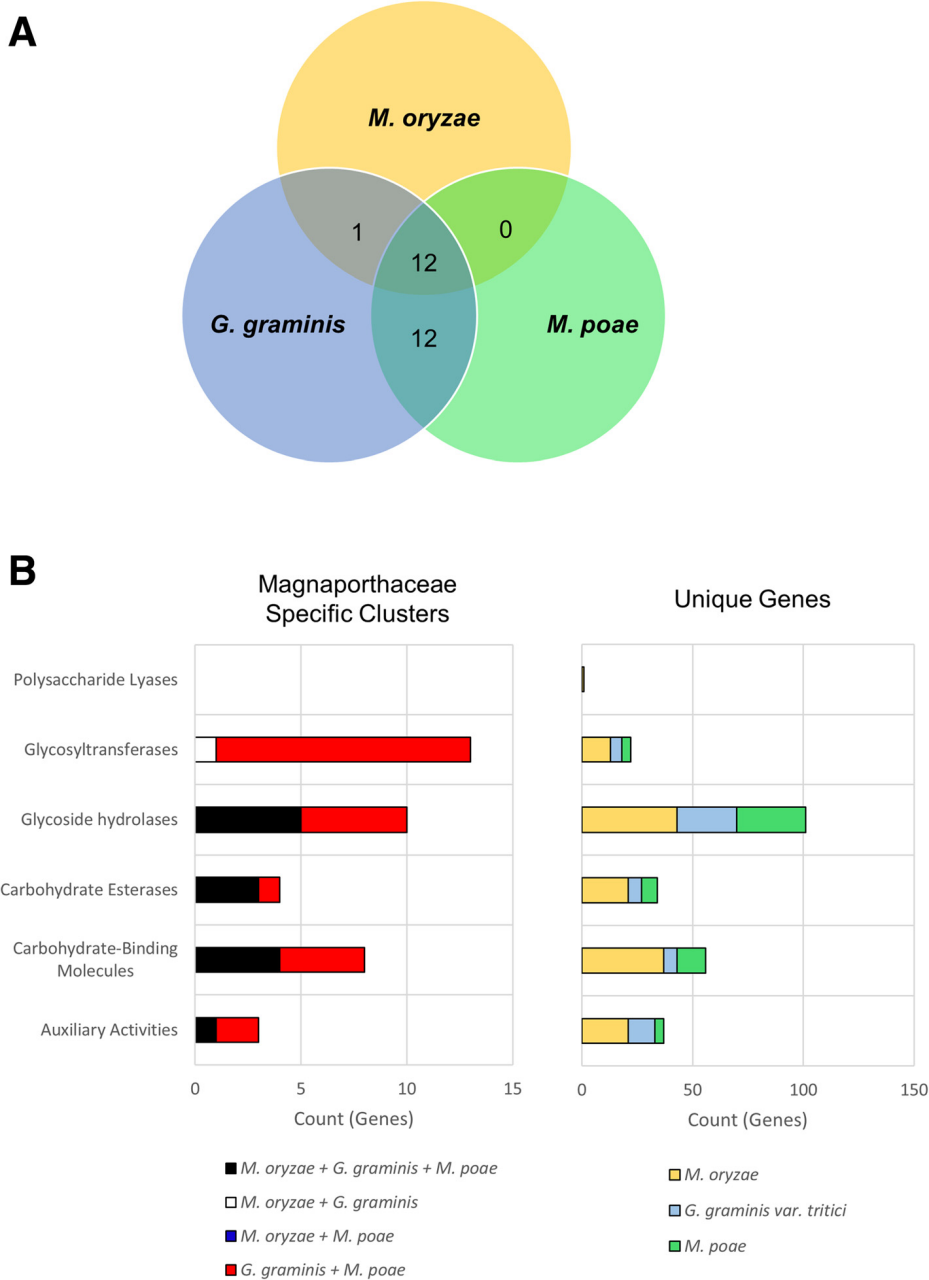
CAZyme analysis of orthologs and unique genes. Hmmscan and dbCAN were used to identify putative carbohydrate active enzymes (CAZymes). **a** Orthologous clusters from OrthoMCL that contained only Magnaporthaceae genes were analyzed. **b** CAZymes were identified by type and counted for Magnaporthaceae specific clusters (*left*) and unique genes (*right*)

Because of the importance of CAZymes in host-pathogen interactions as well as the high number of proteins with enzymatic activity that were identified in our functional analysis (Fig. 2), we utilized Hmmscan 3.0 software [29] with the dbCAN database [30], which enables a more comprehensive analysis than InterProScan, in order to identify OrthoMCL clusters that contained putative CAZymes and the classification of any identified functional domains. Twelve clusters shared between the three species were found to contain putative CAZymes (Fig. 3a). Some clusters were found to contain more than one CAZyme domain classification and are thus represented more than once in Fig. 3b. Five of the twelve clusters contained genes identified as GHs, while four clusters contained CBMs. Fewer clusters were identified as having CEs (3), or auxiliary activity (1), and no clusters were identified as containing GTs or PLs (Fig. 3b, left).

*M. poae* and *G. graminis* var. *tritici* shared twelve clusters with putative CAZyme genes (Fig. 3b, left). *G. graminis* var. *tritici* and *M. poae* shared twelve clusters containing GTs, five clusters containing GHs and four clusters containing CBMs were identified in the *M. poae* and *G. graminis* var. *tritici* shared clusters. *M. oryzae* and *G. graminis* var. *tritici* shared only a single cluster that contained a putative CAZyme, which was identified as containing GTs. Interestingly, *M. poae* and *M. oryzae* had no shared clusters that contained CAZymes.

Analysis of the unique genes for each species revealed that *M. oryzae* had the most unique CAZymes, with 107, while *G. graminis* var. *tritici* and *M. poae* were similar with 50 and 54 unique CAZyme genes, respectively (Fig. 3b, right). The majority of *M. oryzae* CAZymes fell into the GH and CBM categories. For both *M. poae* and *G. graminis* var. *tritici*, GHs were the primary CAZymes identified in the unique genes. Taken together, these data support the previous data by Zhao et al. [9] that glycoside hydrolases are the most abundant CAZymes in the monocot pathogens. These data also show that GTs were abundant in the *M. poae* and *G. graminis* var. *tritici* shared clusters compared with clusters shared by all three Magnaporthaceae species, suggesting that the glycosyltransfereases may be involved in a biological process common to *M. poae* and *G. graminis* var. *tritici*.

### Putative transcription factor identification and analysis

**Fig. 4:**
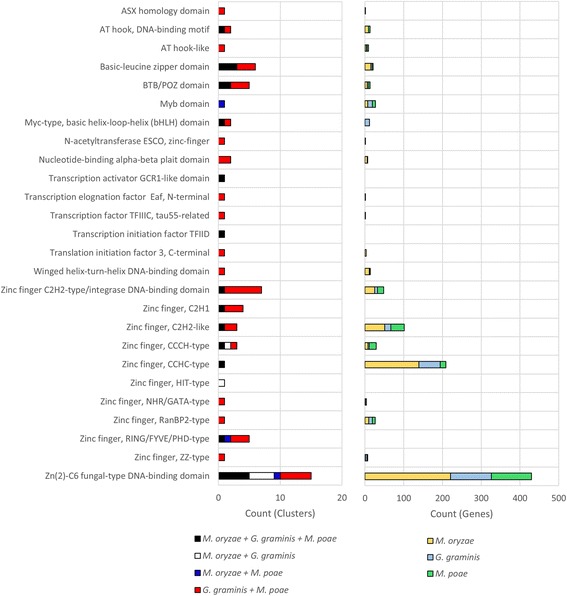
Transcription factor analysis of orthologs and unique genes. InterProScan was used to identify orthologous clusters (*left*) and unique genes (*right*) that had putative transcription factor activity. Custom Python scripts were used to count clusters or genes with each InterPro domain type

One of the more abundant protein types identified in the cluster and unique gene function analysis were proteins with putative transcriptional regulatory activity, including nuclear localization, DNA binding, and transcription factor activity. Using InterProScan to identify specific function domains, we further characterized the putative transcription factors identified in our analyses. In both the Magnaporthaceae specific clusters (Fig. 4, left) and in the unique genes (Fig. 4, right) zinc finger domain containing transcription factors were most abundant. More specifically, the Zn(2)-C6 fungal type DNA binding domain was the most abundant in both data sets, accounting for 15 clusters and over 400 unique genes. Interestingly, the CCHC type zinc finger domain was abundant in the *M. oryzae* unique genes (Fig. 4, right) but not in the *M. poae* or *G. graminis* var. *tritici* unique genes and only account for one cluster containing all three species in the Magnaporthaceae specific OrthoMCL clusters (Fig. 4, left).

### Selection analysis of orthologus clusters

Recent studies have suggested that rapid diversification of certain genes can occur in fungal phytopathogens in response to host plant selection pressures. Mechanisms of increased diversification include proximity to repetitive elements and repeat induced point mutation (RIP), especially in genes close to long-terminal repeat (LTR) retrotransposons [21]. However, most of the studies to date have only been performed in single or small families of genes with similar functions and comparisons were performed in strains of a single species [12, 38–40]. We hypothesized that at the family level, similar patterns would be observed: that genes closer to repetitive elements would exhibit more diversifying selection than genes further from repetitive elements. To test this, orthologous clusters identified by OrthoMCL that contain at least one gene from each Maganporthaceae species were examined for diversifying and purifying selection and their proximity to repetitive elements and putative functions.

**Fig. 5:**
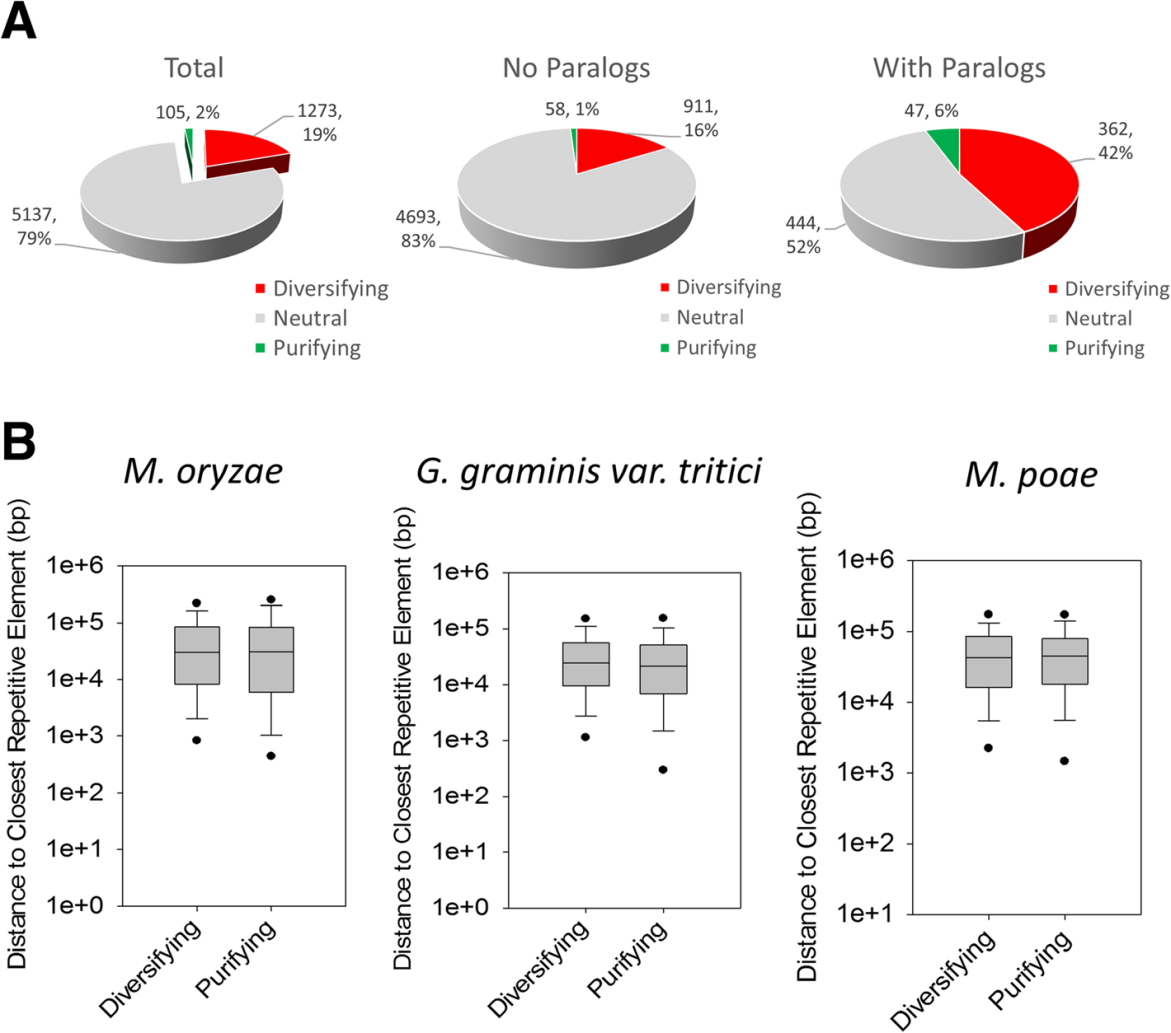
Selection and repetitive element proximity analysis. Clusters containing at least one gene from each species, *M. oryzae*, *G. graminis* var. *tritici*, and *M. poae*, were considered the core proteome. Clusters were analyzed using phylogenetic analysis by maximum likelihood (PAML) and determined to be under diversifying, purifying, or neutral selection. **a** Proportions of diversifying (*red*), purifying (*green*), and neutral (*grey*) clusters compared to the total number of clusters (*left*) were graphed. Clusters containing only a single gene from each species were parsed and proportions of diversifying, purifying, and neutral clusters were graphed (*middle*). Clusters containing more than one gene for at least one species were also parsed and graphed (*right*). **b** Distance to the closest repetitive element was identified for genes in diversifying and purifying clusters for *M. oryzae* (*left*), *G. graminis* var. *tritici* (*middle*), and *M. poae* (*right*). *Black dots* represent 5^th^ and 95^th^ percentiles. *p*-values were calculated using the Mann—Whitney Rank Sum test

Six-thousand five-hundred eighteen clusters which contained genes from all three Magnaporthaceae species were considered the “core proteome” and were used for further analysis. Core proteome clusters were subjected to phylogenetic analysis by maximum likelihood (PAML) [31, 41] using CODEML, an algorithm within PAML. Rates for non-synonomous (dN) and synonomous (dS) mutations were calculated and clusters with a dN/dS ratio greater than one that were statistically significant and met best fit models within CODEML were considered to be under diversifying selection, while those clusters with dN/dS ratios less than one that were statistically significant and met best fit models within CODEML were considered to be under purifying selection. Of the core proteome clusters, 79 % were found to be under neutral selection (Fig. 5a, left), while 19 % were under diversifying selection and 2 % were under purifying selection.

The vast majority of core proteome clusters (87 %) were found to contain a single gene from each of the three Magnaporthaceae species while only 13 % contained paralogs. We hypothesized that the clusters that contained paralogs were undergoing more diversifying selection than those with a single gene from each species. To test this, the clusters were split into two categories, those with a single gene from each species (Fig. 5a, middle, No Paralogs), and those that contain putative paralogs (Fig. 5a, right, With Paralogs). We observed that fewer clusters containing paralogs were under neutral selection. In addition, both the proportion of clusters under purifying selection (6 %) and diversifying selection (42 %) were higher compared with the clusters with no paralogs. Thus, clusters that contain paralogs are under more selection than those without paralogs, but the selection is not limited to purifying or diversifying.

Repetitive element sequence analyses in several fungal species has been used to identify evolutionary relationships between species based on repetitive element copy number and location. Hypotheses have been suggested that genomes can evolve at two different speeds due to proximity to and influence by repetitive elements, where diversifying genes are in regions of high repetitive content, while conserved genes are in area with low repetitive content. Previous studies have shown a high mutation rate due to repeat induces point mutation (RIP) in areas of the *M. oryzae* genome which contain specific long-terminal repeat (LTR) retrotransposons, such as Maggy [21]. Therefore, PAML scores for the core proteome were compared to repetitive element content of the DNA near each gene.

Briefly, repetitive element libraries were built for each Magnaporthaceae species de novo using RepeatModeler [36, 42]. Only repetitive elements >200 bp were considered for further analysis. For each species, genes were identified as undergoing diversifying, or purifying selection and their distances to the closest repetitive element were graphed (Fig. 5b). *P*-values were then calculated using the Mann—Whitney Rank Sum test comparing the diversifying gene group and the purifying gene group to determine if there is a significant difference in the distance between repetitive elements for each group. *P*-values of <0.05 were considered statistically significant. Surprisingly, there was no significant difference between the distance to the closest repetitive element between diversifying and purifying genes for *M. oryzae* (*p* = 0.128), *G. graminis* var. *tritici* (*p* = 0.756), or *M. poae* (*p* = 0.580). Taken together, these data do not support our hypothesis but rather suggest that there is no correlation between proximity to repetitive elements and diversifying or purifying selection.

**Fig. 6:**
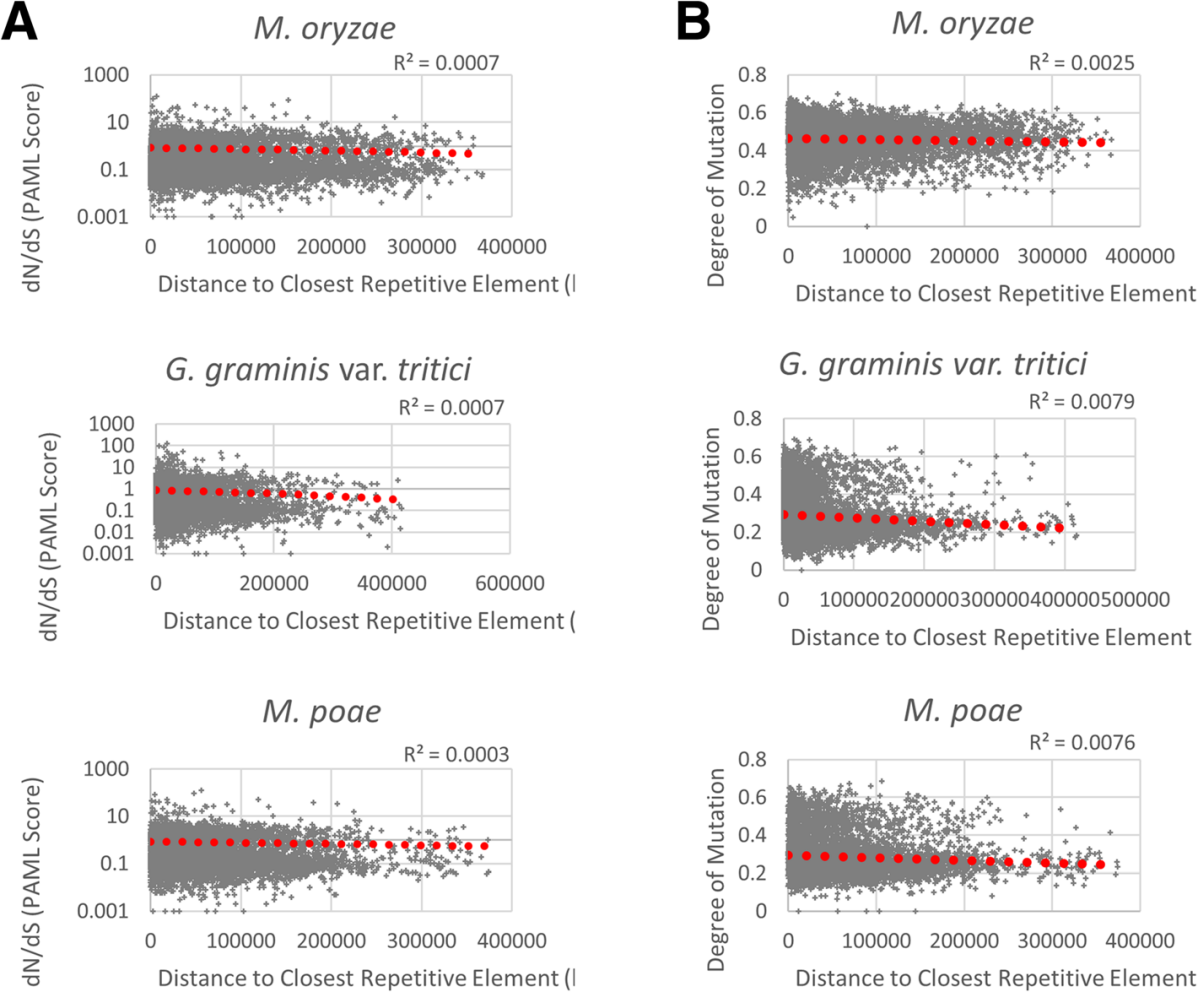
Core proteome and repetitive element proximity. **a** PAML scores for individual genes were graphed against the distance to the closest repetitive element for *M. oryzae* (*top*), *G. graminis* var. *tritici* (*middle*), and *M. poae* (*bottom*). *Red dotted lines* indicate linear regression equations. **b** The genes of the core proteome was analyzed for degree of mutation, where higher values indication more accumulated mutations. Degree of mutation was graphed against the distance to the closest repetitive element for *M. oryzae* (*top*), *G. graminis* var. *tritici* (*middle*), and *M. poae* (*bottom*). *Red dotted lines* indicate linear regression equations

In order to confirm the observation that there is no effect of distance to repetitive elements and diversifying or purifying selection on genes within the Magnaporthaceae family, the genes that made up the core proteome were graphed as the dN/dS ratio for the cluster versus the gene’s distance to the closest repetitive element and coefficient of determination (R^2^ values) were calculated (Fig. 6a). For all three Magnaporthaceae species, the R^2^ value was near zero and ranged from 0.0003 for *M. poae* to 0.0007 for *M. oryzae* and *G. graminis* var. *tritici*. These data suggest that there is no correlation between PAML score and closest repetitive element.

dN/dS ratio does not take into account the total number of mutations found in a gene sequence, therefore, additional mutational analysis was performed. Briefly, mutational analysis was performed by predicting a majority consensus sequence for each sequence and then identity distances between the consensus and each sequence in the alignments were calculated using the majority character at each site. For each gene sequence in the ortholog cluster, the pairwise distance between the consensus and the transcript sequence were calculated. Values ranged from 0 to 0.69, hereby regarded as the degree of mutation, with the values closer to one representing genes with the highest proportion of total mutations. Degree of mutation for each gene in the core proteome was graphed against the distance to the closest repetitive element and coefficient of determination (R^2^ values) were calculated in order to determine if there was a correlation between degree of mutation and repetitive element proximity (Fig. 6a). The R^2^ values were near zero for *M. oryzae* (R^2^ = 0.0025), *G. graminis* var. *tritici* (R^2^ = 0.0079) and *M. poae* (R^2^ = 0.0076). These data suggest that for the orthologous clusters within the Magnaporthaceae family of fungi, the degree of mutation is not correlated with the distance to the closest repetitive element.

### Identification of function for diversifying and purifying gene clusters

**Fig. 7:**
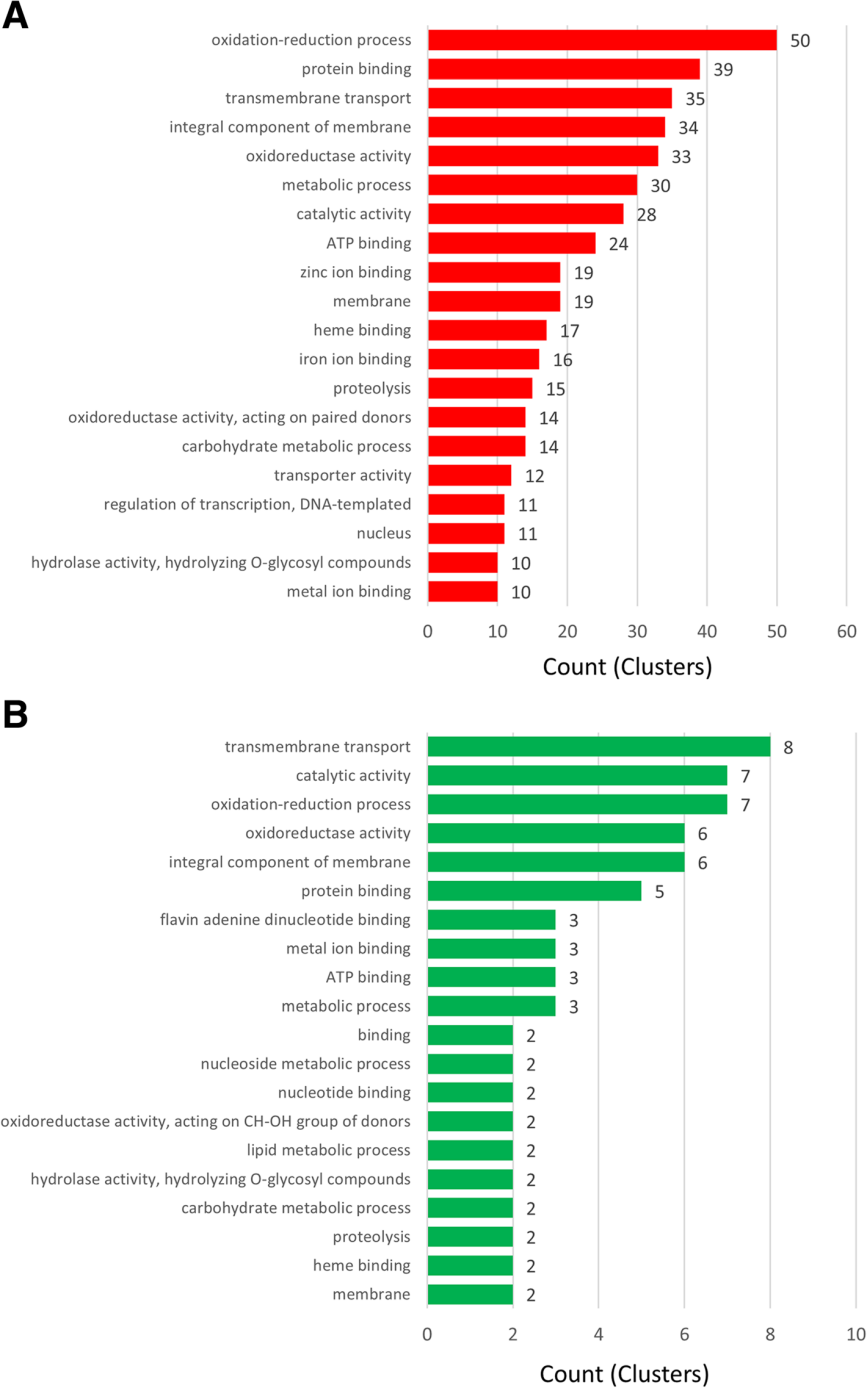
Functions for diversifying and purifying clusters. Putative functions were identified using GO annotation within InterProScan. The most abundant 20 GO categories were graphed for the **a** diversifying clusters (*red*) and **b** purifying clusters (*green*)

While no overall relationship between PAML score or degree of mutation and repetitive DNA content was observed in any of the three species of Magnapothaceae, we wanted to identify the functions for genes that exhibit diversifying selection or purifying selection. Genes within the clusters undergoing diversifying or purifying selection were subjected to GO annotation to determine putative function. Approximately 55 % of diversifying clusters and 38 % of purifying clusters had no GO annotation. The most abundant twenty GO categories were graphed for diversifying clusters (Fig. 7, Top) and purifying clusters (Fig. 7, bottom). Interestingly 14 of the 20 categories were the same between the diversifying and purifying clusters, suggesting that genes in these categories are under selection. However, binding, nucleotide binding, and nucleoside and lipid metabolic processes were represented in the purifying clusters and not in the diversifying clusters. In contrast, regulation of transcription, nucleus, and zinc binding were all represented in the diversifying clusters. These data suggest that binding and some subsets of metabolism are conserved while transcription and ion binding are not. Together with the observation that zinc binding transcription factors are abundant in both the Magnaporthaceae specific OrthoMCL clusters and genes unique to each of the three fungi (Fig. 2, Fig. 4), these data suggest a role for transcription factors in speciation within the Magnaporthaceae family of fungi.

### Secreted protein identification and analysis

Several secreted proteins in *M. oryzae* have been identified as effector proteins, which play a role in modulating the host immune response to infection (reviewed in [43]). It has been proposed that such effector proteins must be more prone to mutation than the rest of the fungal genome in order to evade host plant recognition and defenses [10, 11]. These studies suggest that small secreted proteins, defined here as under 250 amino acids in length, may be undergoing diversification due to close proximity to repetitive elements. Because our data show no correlation between diversifying selection and proximity to repetitive elements at the genome level, the relationship between small secreted proteins and repetitive element location was examined.

**Fig. 8:**
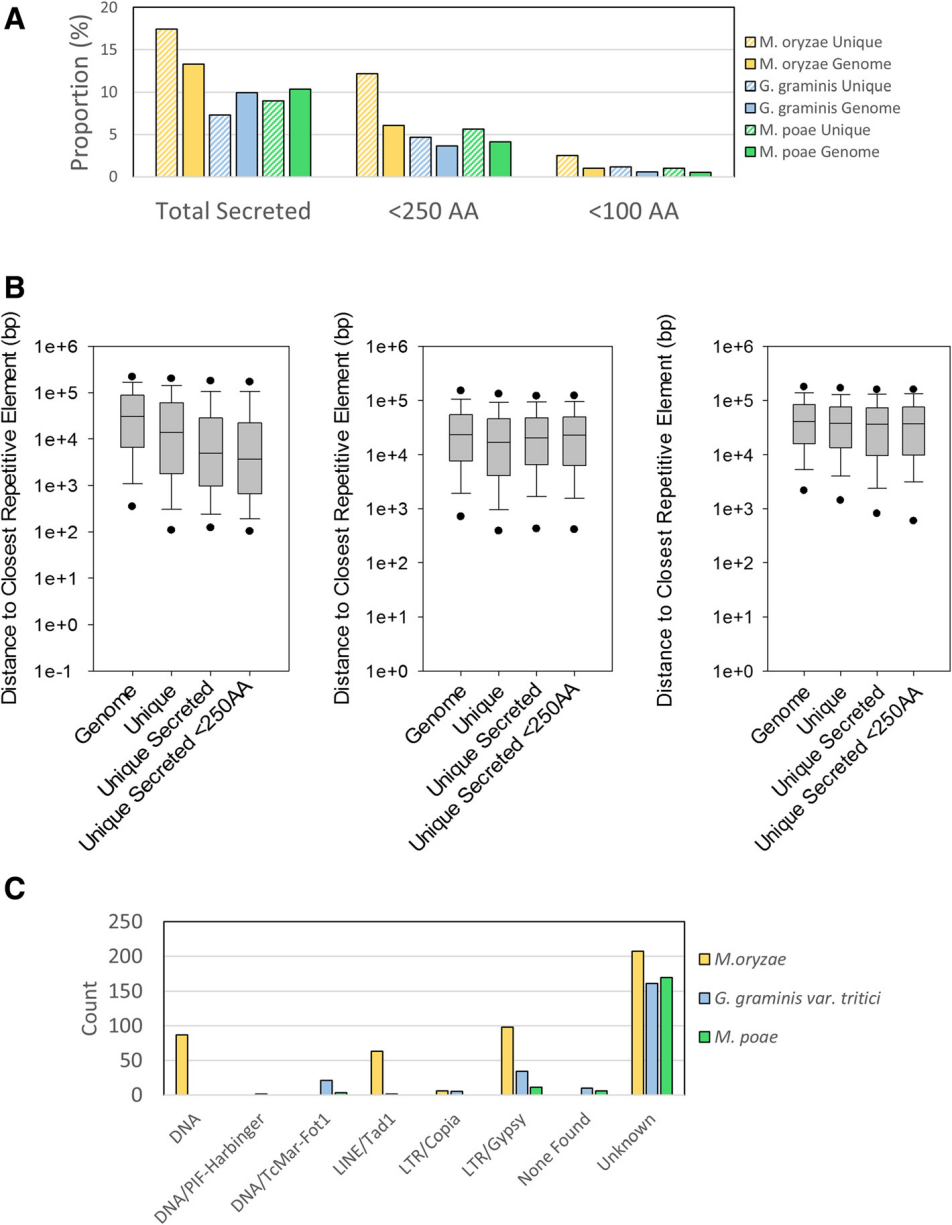
Secreted protein identification and analysis. Targetp and Signalp were used to identify secreted proteins in the Magnaporthaceae species. **a** The total proportion of secreted proteins that are unique to each species compared with the proportion of secreted proteins in the whole genome (*left*), the proportion of unique secreted proteins under 250 amino acids in length compared to the proportion of secreted proteins under 250 amino acids in length in the whole genome (*middle*), and the proportion of unique secreted proteins under 100 amino acids in length compared to the proportion of secreted proteins under 100 amino acids in length in the whole genome (*right*). **b** Distance to the closest repetitive element was graphed for the whole genome (genome), total unique genes (unique), all unique secreted genes (unique secreted), and unique secreted genes less than 250 amino acids in length (unique secreted <250AA) for *M. oryzae* (*left*), *G. graminis* var. *tritici* (*middle*), and *M. poae* (*right*). *Black dots* indicate 5^th^ and 95^th^ percentile. **c** The classifications for the closest repetitive elements for the small secreted proteins (<250AA) were counted for each species

TargetP [32] and SignalP [33, 44] were used to identify proteins that contained signal sequences and are targeted to the secretory pathway. *M. oryzae* contained the highest proportion of secreted proteins with approximately 13 % of the proteins in the genome containing such signal sequences (Fig. 8a). In contrast, 10 % of the genome was identified as secreted proteins in both *G. graminis* var. *tritici* and *M. poae*. In addition to the whole genome, secreted proteins were identified among the genes unique to each species. In *M. oryzae*, there was an enrichment of secreted proteins among the unique genes, with 17 % of the unique gene population identified as secreted proteins compared with the whole genome, which contains roughly 13 % secreted proteins. In contrast, 7 % of the *G. graminis* var. *tritici* unique genes were secreted proteins and 9 % of *M. poae* unique genes were secreted proteins. All three species of Magnaporthaceae showed an enrichment of unique secreted proteins less than 250 amino acids in length compared with the proportion of secreted proteins under 250 amino acids in length found in the whole genome. However, the enrichment was exaggerated in *M. oryzae*, which has 12 % unique secreted proteins less than 250 amino acids compared with 6 % secreted proteins less than 250 amino acids in the total genome (Fig. 8a). A similar trend was observed when the cutoff for small secreted proteins was changed to 100 amino acids.

Because it has been proposed that small secreted proteins undergo faster evolution due to proximity to repetitive elements, the distance between unique proteins (UP), unique secreted proteins (USP), and unique secreted proteins smaller than 250 amino acids (USP250) was compared to the closest repetitive elements (Fig. 8b). In *M. oryzae*, UP, USP, and USP250 were significantly closer to repetitive elements when compared with the genome average (*p* < 0.001 for all comparisons). In addition, USPs were significantly closer to repetitive elements than UP (*p* < 0.001). However, there was no significant difference between the USPs and USP250s (*p* = 0.054). Interestingly, this trend was only observed in *M. oryzae*. In *G. graminis* var. *tritici* and *M. poae*, only UP has a significant difference when compared with the total genome (*p* < 0.001 and *p* = 0.008, respectively). There was no significant difference observed in the USP or USP250 in *G. graminis* var. *tritici* or *M. poae* when compared with the whole genome average.

High mutation rates and C–G → A–T point mutations are found to be associated with certain retrotransposons in *M. oryzae* [21]. Therefore, we examined the closest repetitive elements to the USP250 to identify the subtype. We observed that in all three Magnaporthaceae, repetitive elements that were classified as “unknown” by RepeatModeler were most commonly found with small secreted proteins (Fig. 8c). A small proportion of *G. graminis* var. *tritici* and *M. poae* USP250 have no repetitive elements mapped to the same contig and were unable to be fully analyzed (Fig. 8c, None Found). Of the identified repetitive elements, retrotransposons were most commonly identified as the closest repetitive element to the USPs, with LTR/Gypsy and LINE/Tad1 elements being highly represented in *M. oryzae* (Fig. 8c). Thus, these data suggest that retrotransposons are the closest repetitive elements the small secreted proteins in *M. oryzae*. However, these observations cannot be extrapolated to *M. poae* or *G. graminis* var. *tritici*.

**Fig. 9:**
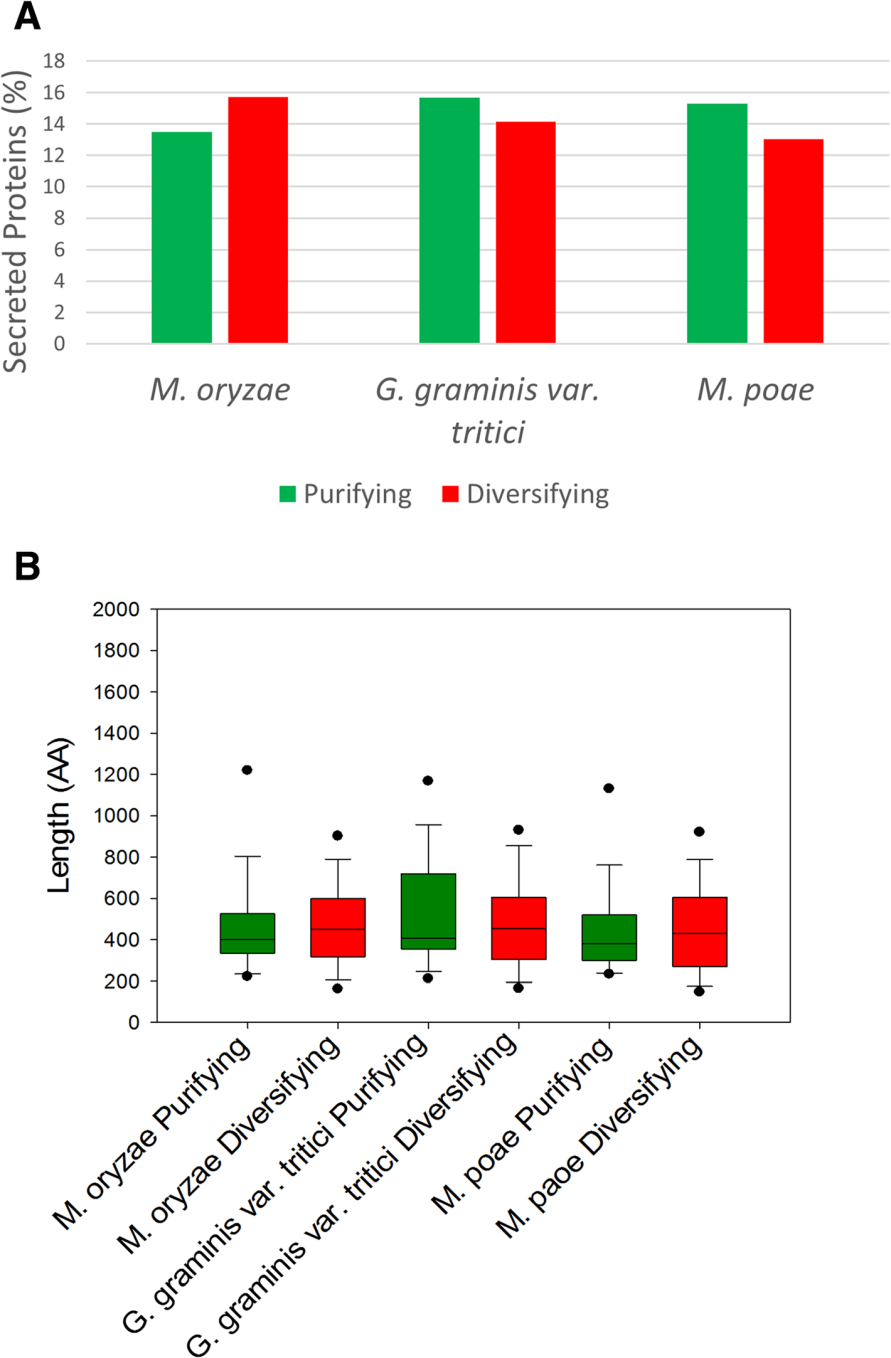
Purifying and diversifying selection on secreted proteins. Proteins undergoing purifying or diversifying selection were identified using TargetP and SignalP. **a** The proportion of purifying (*green*) or diversifying (*red*) proteins that were identified as secreted proteins for the three Magnaporthaceae species. **b** The length of secreted protein identified in the purifying (*green*) and diversifying (*red*) clusters. *Black dots* indicate 5^th^ and 95^th^ percentile

Genes identified as putative secreted proteins within purifying and diversifying clusters were analyzed further. The proportion of purifying and diversifying genes that are secreted were graphed and the *p*-value was calculated comparing purifying and diversifying genes within each Magnaporthaceae species (Fig. 9a). There was no significant difference between the proportion of secreted proteins undergoing purifying selection and the proportion of secreted proteins undergoing diversifying selection (*M. oryzae p* = 0.2278, *G. graminis* var. *tritici p* = 0.2884, and *M. poae p* = 0.205). In addition, there was no significant difference in the length of secreted proteins (Fig. 9b) that undergoing purifying selection compared with secreted proteins that are undergoing diversifying selection (*M. oryzae p* = 0.563, *G. graminis* var. *tritici p* = 0.790, and *M. poae p* = 0.788). While there is evidence that small secreted proteins are closer to repetitive elements in the genome, these data suggest that neither secreted proteins nor small secreted proteins are enriched in the diversifying clusters compared with the purifying clusters. Thus, repetitive element proximity does not appear to influence purifying or diversifying selection.

## Discussion

The Magnaporthaceae family of fungi is both economically and socially important; understanding the infection process and identifying novel antifungal targets are becoming critical to halt widespread crop and turf grass loss. Here we utilized several analytical approaches to interrogate conserved and unique genes among three species of Magnaporthaceae. Using OrthoMCL, we identified clusters that are highly conserved among 74 fungal species and 1149 clusters that are specific to the Magnaporthaceae (Fig. 1). In addition, we identified which genes are unique to each species and determined putative gene functions (Table 1, Fig. 2, Fig. 3, and Fig. 4). OrthoMCL revealed a core proteome for the Magnaporthaceae of 6518 clusters that contain at least one gene from *M. oryzae*, *G. graminis* var. *tritici*, and *M. poae*. To our surprise, further analysis of the core proteome using PAML revealed that there is no correlation between PAML score and distance to repetitive elements (Fig. 6a) or degree of mutation to repetitive elements (Fig. 6b), while analysis of clusters that are undergoing diversifying or purifying selection showed no enrichment of secreted proteins nor small secreted proteins (Fig. 9a, b).

GO annotation and InterProScan analysis of the clusters unique to the Magnaporthaceae species showed that proteins with enzymatic function and proteins involved in transcriptional regulation were the most common. However, these categories are also common in the genes that are unique to each species. These data suggest that both categories may contribute to speciation but not enough evolutionary time has passed to separate the genes in the shared clusters into unique genes. Alternatively, there may be some evolutionary pressure to maintain the genes within the 295 shared clusters, such as environmental conditions or host plant conditions. Putative function analysis was performed at the cluster level, thus leaving the potential that analysis at the sequence level will reveal specific conserved regions of the genes within each cluster.

Interestingly, more CAZymes were found to be specific to each species compared with the Magnaporthaceae specific clusters (Fig. 3). These data suggest that CAZyme gene sequences are plastic and may contribute to speciation. Fungi produce a large number of CAZymes [9] and an abundance of proteins with redundant functions may result in the diversity observed in our data. Zhao et al. [9] showed that there were similar ratios of each CAZyme class found in fungi that infect similar hosts, such as monocots or dicots. However, CAZymes may vary based on route of infection rather than the type of host plant. *M. oryzae* infects the leaf of a plant while *G. graminis* var*. tritici* and *M. poae* infect the root of the plant. *G. graminis* and *M. poae* share 12 clusters that contain CAZymes that are not shared with *M. oryzae* (Fig. 3a). One hypothesis for the abundance of shared CAZymes is that these clusters contain genes needed to infect the root of the host plant. Our CAZyme analysis suggests that glycosyltransferases may be important in the environmental or host-pathogen interactions in *M. poae* and *G. graminis* var. *tritici*, while glycoside hydrolases and carbohydrate binding molecules are most abundant among the *M. oryzae* unique genes (Fig. 3b). Further analysis of the CAZyme families may reveal specific enzyme targets for each cluster that are important to infection at the root or leaf.

In addition to enzymes, transcription factors were identified as abundant in both the Magnaporthaceae specific clusters and the unique gene groups for each species (Fig. 4). More specifically, the zinc finger and fungal-type zinc finger transcription factors were common in both analyses. These data suggest that adaptation to environmental and host-plant stresses may be dependent on transcriptional regulation in addition to altering protein function through mutation. Preliminary RNAseq data of the three species under several stress conditions, such as heat, cold, and osmotic stress, suggest that relatively few clusters exhibit similar transcriptional regulation (data not shown), however, additional experiments must be performed to confirm these data.

The ratio of purifying and diversifying clusters compared with clusters under neutral selection varied depending on the presence or absence of paralogs (Fig. 5a). In clusters that contained one or more paralogs, there was an increase in the proportions of both diversifying and purifying genes. There are several proposed functions for gene duplication in fungi. First, gene duplication of genes with highly conserved function (purifying genes) may be needed to maintain genes with redundant function. Second, duplication of conserved genes may result in increased protein production. Third, gene duplication of diversifying proteins may be needed to develop a novel function for the gene group. Our data suggests that the gene duplication observed in the core proteome results in both conserved and novel functions. Closer analysis of clusters and their function would be needed to further understand the nature of each gene duplication.

It has been suggested that in *M. oryzae*, genes encoding effectors are undergoing more rapid evolution than other genes [12–15, 22]. As hypothesized for antagonistic co-evolution between organisms, the zig-zag model of host and pathogen evolution suggests that as the host immune system evolves to recognize certain pathogen effector proteins, then the pathogen must, in turn, evolve to evade the host immune response [11, 45]. The avirulence genes (AVR) in *M. oryzae*, *Leptosphaeria maculans*, *Leptospheraeria biglobosa*, and other phytopathogenic fungi, have been shown to have undergone gene duplication, translocation, and RIP mutation [3, 6, 11–24], supporting the idea that these effector proteins are undergoing rapid mutation. Interestingly, the zig-zag model of evolution between host and pathogen is not limited to fungal pathogens nor plant hosts, but is also seen in a variety of host-pathogen interactions such as mammalian parasitic pathogens including the malaria causing *Plasmodium falciparum* [46]. The merozoite surface protein I (MSP1) gene in *P. falciparum* is highly polymorphic, allowing for evasion of the host antibody response [46].

Additionally, it has been suggested that proximity to repetitive elements, such as retrotransposons, contributes to rapid diversification [12–22]. More specifically, the *M. oryzae* LTR retrotransposon, Maggy, has been found to be associated with T:A enriched regions due to RIP [21]. Our data does show that in *M. oryzae* unique proteins and, more specifically, small unique proteins are closer to repetitive elements, including LTR classification (Fig. 8b, c). However, these observations were not seen in either *G. graminis* var. *tritici* or in *M. poae*, suggesting that increased diversification due to repetitive element proximity, and more specifically proximity to Maggy and similar retrotransposons, is not universal to the Magnaporthaceae family of fungi.

It is important to note that the purpose of this study was to compare three genomes of related phytopathogenic fungi at the family level. While our data shows no evidence of a two-speed genome evolution in the Magnaporthaceae, evidence of small scale evolution, such as diversification observed between strains, may still be found. While we hypothesized that evidence of a two-speed genome evolution would be observed among the Magnaporthaceae family, our analyses, which were performed in several different ways (Figs. 6a, b, 8a–c, 9a, b) failed to support the hypothesis.

Our data showed that at the genome level, there is no evidence to suggest multi-speed genome evolution or that proximity to repetitive elements plays a role in diversification of genes. Our core proteome analysis consisted of 6518 clusters containing a total of 22,085 genes from *M. oryzae*, *G. graminis* var. *tritici*, and *M. poae*. We examined the proximity of genes undergoing diversifying or purifying selection to repetitive elements and determined there was no significant difference between the two groups in any species (Fig. 5b). To confirm these data, PAML scores were graphed against distance to repetitive elements, R^2^ values were near zero (Fig. 6a) and mutation analysis (Fig. 6b) also confirmed no correlation between degree of mutation and proximity to repetitive elements. Because sequence homology is used to cluster orthologs in OrthoMCL, it is possible that more conserved genes were used in our analysis. Thus by comparing orthologs, the data may be skewed towards neutral or purifying clusters. However, by using a low cutoff of 50 % sequence homology implemented in OrthoMCL to cluster orthologs, clustering should include a wider range of diversified genes.

## Conclusions

Taken together, our data suggests that there is no evidence for two-speed evolution at the genome level. Additionally, repetitive element proximity has no influence on diversification of purification of orthologous clusters. While it is possible for more rapid evolution can occur on a small scale, such as a small group or functional class of proteins, these trends cannot be observed at the genome level.

## Methods

### Genome sequences and OrthoMCL

Genome, transcript, and protein sequences for 74 fungal genomes were downloaded from the Fungal Genome Initiative at Broad Institute of Harvard and the Massachusetts Institute of Technology [34]. A comprehensive list of the source files used can be found in Additional file 1. A phylogenetic tree representing the 74 fungal genomes was made using phylo T [47] and can be found in Additional file 2. For OrthoMCL analysis [1, 2] the protein sequences from 74 completed fungal genomes (including *M. oryzae*, *M. poae*, and *G. graminis* var. *tritici*) were compared using BLASTp (all-vs-all) with a maximum E-value of 1e–5. From the resulting BLASTp hits OrthoMCL identified homologous and paralogous relationships at 50 % similarity. Markov clustering was used to further refine orthologous clusters as described previously [2]. Orthologous clusters can be found in Additional file 3. Three criteria were used to identify genes considered unique to each species: genes that were excluded from OrthoMCL clustering after all-vs-all BLASTp analysis, genes that were not clustered during Markov clustering, and all genes within clusters containing a single species.

### Gene and cluster functions

Putative cluster functions were identified using the Blast2GO [37] suite of software, including BLASTn, InterPro protein domain identification, Gene Ontology annotation with *Aspergillus* slim. InterProScan v5.14 software [28] was used to determine the functions of unique genes. Functional domains from protein sequence files [34] were identified using PROSITE, HAMAP, Pfam, PRINTS, ProDom, SMART, TIGRFAMs, PIRSF, SUPERFAMILY, CATH-Gene3D, and PANTHER protein databases through Blast2GO [37] and InterProScan [28]. Gene Ontology (GO) terms were identified using InterProScan [27, 28].

### CAZyme identification and classification

OrthoMCL clusters that were specific to the Magnaporthaceae were searched for carbohydrate activity enzymes (CAZymes). Fungal specific CAZymes were identified in the Magnaporthaceae protein sequences using Hmmscan v3.1b2 [29] and dbCAN v4.0 [30] database. Output files were parsed using the parser perl script included in the dbCAN database.

### Transcription factor identification and classification

Conserved transcription factors were identified using InterProScan v5.0 software domain identification [28]. Functional domains predicted by InterProScan analysis were used to identify putative transcription factors. Custom Python v3.4 [48] scripts were used to parse and count putative transcription factors. InterProScan output data was manually inspected for genes with putative transcription factor analysis to ensure that all transcription factors were identified and no extraneous genes were included.

### Phylogenetic analysis by maximum likelihood

OrthoMCL clusters that contained at least one gene from each Magnaporthaceae species were parsed and transcripts for genes within each cluster were retrieved from the Broad Institute transcript files using custom Python scripts. The paired sequence files were aligned using command line MUSCLE v3.8.31 [49], reiterating the alignments until reaching convergence. Phylogenetic trees were simultaneously generated from the second iteration. Alignment columns with more than 65 % gap characters were removed using a custom Python script. Three clusters (moggtmp1004, moggtmp1005, and moggtmp1315) were unable to be aligned and were not analyzed further. In order to estimate the nonsynonymous to synonymous (dN/dS) substitution rates, the CODEML program as part of PAML v4.8 [31] was implemented using BioPython v1.65 [50]. Likelihood ratio tests (LRTs) of site-specific selection were used, comparing M1 (neutral) to M2 (selection) and M7 (beta) to M8 (beta & w) using the test statistic 2*(lnL1–lnL2) = 2∆L. The cluster was considered undergoing positive selection if both the M1/M2 and M7/M8 LRTs were significant under a chi-square test with *p* < 0.05.

### Repetitive elements identification and classification

Repetitive elements were identified as previously described [36]. Briefly, repetitive element analysis was performed using RepeatModeler and RepeatMasker programs [45]. *De novo* repetitive element libraries were created with RMBlast NCBI search engine within RepeatModeler. Similar repetitive element sequences were collapsed into their parent family and classified within RepeatModeler. Final classified consensus files for *M. poae* and *G. graminis* var. *tritici* were used as libraries for repetitive element searches with RepeatMasker. Repetitive sequence larger than 200 bp were considered for further analysis. Custom perl scripts were used to determine the distance to right flanking and left flanking repetitive element for each gene in the genomes of each of the three Magnaporthaceae. Box plots were graphed and Mann—Whitney Rank Sum statistical tests were performed using SigmaPlot v12.5 [51].

Mutational analysis was performed by predicting a majority consensus sequence for each sequence, using the seqinr v3.1-1 package incorporated into R. The identity distances between the consensus and each sequence in the alignments were calculated using the majority character at each site. The pairwise distance between the consensus and the transcript sequences were calculated. The degree of mutation was calculated as the squared root of the identity between the consensus and sequence.

### Secreted protein identification and analysis

In order to identify secreted proteins, a two-step process was used; first protein sequences that contained a signal sequence were identified, then the subcellular localization of each was determined. Sequences that contained both a signal sequence and were identified as being targeted to the secretory pathway were considered secreted proteins. To identify proteins containing signal sequences, whole genome protein sequence files were analyzed using SignalP v4.1 [33]. Those protein sequences that were identified as having a signal sequence by SignalP were then analyzed by TargetP v1.1 [32]. VasserStats [52] was used to determine Z-scores and *p*-values for proportions. Genes from clusters identifies as undergoing purifying or diversifying selection by PAML analysis were analyzed for secreted proteins using SignalP and TargetP. Protein lengths for identified secreted proteins were graphed as box plots and Mann—Whitney Rank Sum statistical tests were performed with SigmaPlot v12.5.

## Availability of supporting data and materials

All genome and protein sequence files are available through GenBank (http://www.ncbi.nlm.nih.gov/genbank/) and FungiDB (http://www.fungidb.org) and are noted in Additional file 1. OrthoMCL output data is available in Additional file 3.

## Additional files

Additional file 1:
**List of genomes analyzed by OrthoMCL.** (XLSX 14 kb) Additional file 2:
**Phylogenetic tree of species analyzed by OrthoMCL.** 74 fungal genomes from the Broad Institute were compared using OrthoMCL. The genomes consisted of plant pathogens, mammalian pathogens, and model organisms (TIF 1348 kb) Additional file 3:
**OrthoMCL clusters.** (TXT 8405 kb)
