# Supplementary figures and images for: Comparative genome analysis and genome evolution of members of the magnaporthaceae family of fungi
Source: BMC Genomics. 2016 Feb 25;17:135. doi: 10.1186/s12864-016-2491-y (PMC4766678; doi:10.1186/s12864-016-2491-y)

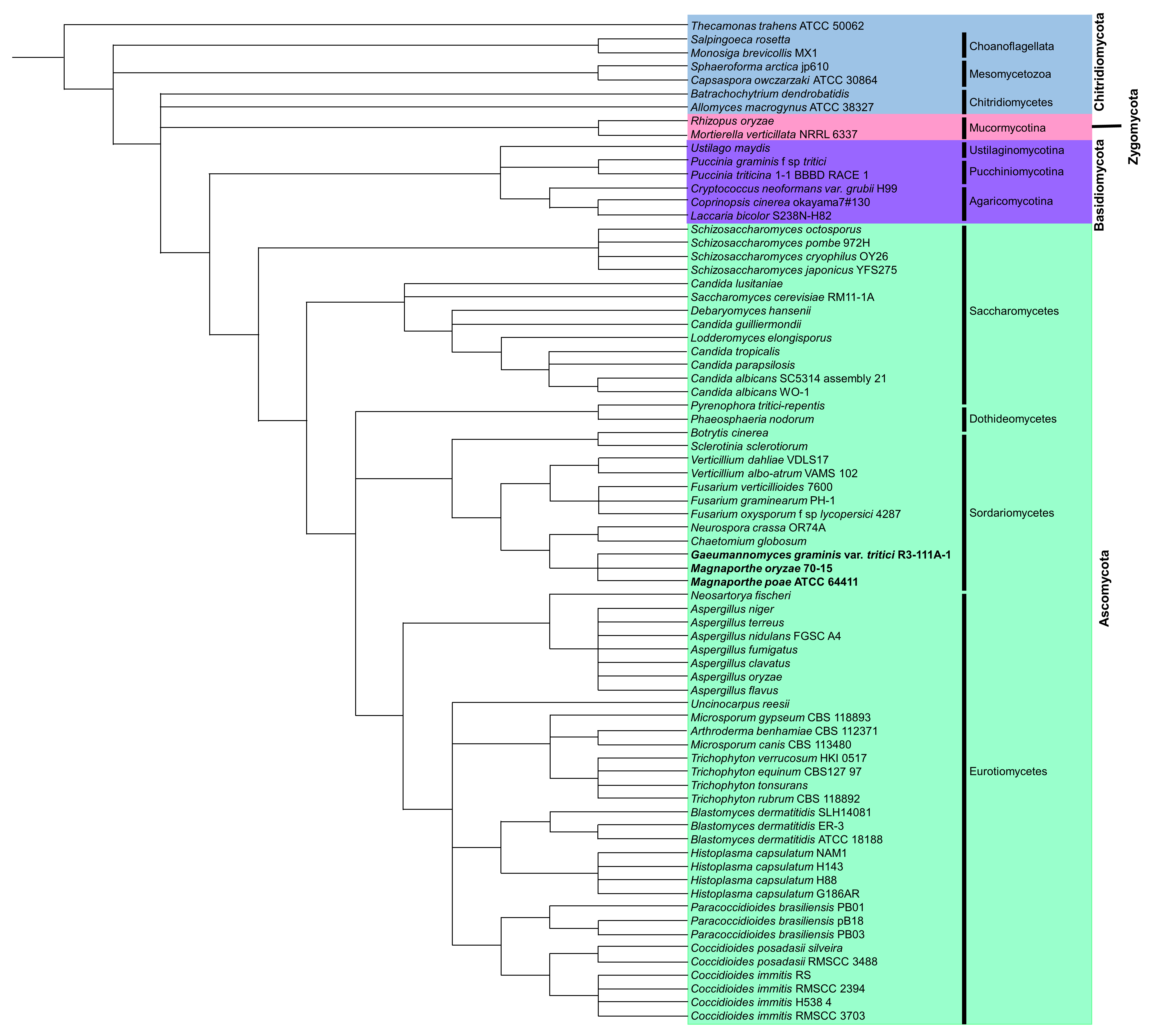

Supplement: Additional file 2: — Phylogenetic tree of species analyzed by OrthoMCL. 74 fungal genomes from the Broad Institute were compared using OrthoMCL. The genomes consisted of plant pathogens, mammalian pathogens, and model organisms (TIF 1348 kb) [file 12864_2016_2491_MOESM2_ESM.tif]
